# Intracellular Protein–Lipid Interactions Studied by Rapid-Scan Electron Paramagnetic Resonance Spectroscopy

**DOI:** 10.1021/acs.jpclett.0c03583

**Published:** 2021-03-05

**Authors:** Theresa S. Braun, Juliane Stehle, Sylwia Kacprzak, Patrick Carl, Peter Höfer, Vinod Subramaniam, Malte Drescher

**Affiliations:** † Department of Chemistry and Konstanz Research School Chemical Biology, University of Konstanz, 78457 Konstanz, Germany; # Bruker BioSpin GmbH, Silberstreifen 4, 76287 Rheinstetten, Germany; § Vrije Universiteit Amsterdam, De Boelelaan 1105, 1081 HV Amsterdam, The Netherlands

## Abstract

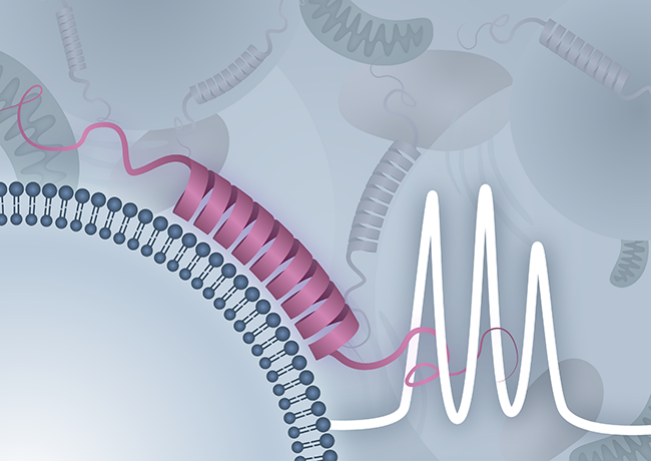

Protein–membrane interactions play key roles in essential cellular processes; studying these interactions in the cell is a challenging task of modern biophysical chemistry. A prominent example is the interaction of human α-synuclein (αS) with negatively charged membranes. It has been well-studied *in vitro*, but in spite of the huge amount of lipid membranes in the crowded environment of biological cells, to date, no interactions have been detected in cells. Here, we use rapid-scan (RS) electron paramagnetic resonance (EPR) spectroscopy to study αS interactions with negatively charged vesicles *in vitro* and upon transfection of the protein and lipid vesicles into model cells, *i.e.*, oocytes of *Xenopus laevis*. We show that protein–vesicle interactions are reflected in RS spectra *in vitro* and in cells, which enables time-resolved monitoring of protein–membrane interaction upon transfection into cells. Our data suggest binding of a small fraction of αS to endogenous membranes.

The interaction of proteins with endogenous membranes is a fundamental feature for regulation of various intracellular processes. The binding of specific lipids in the cell membrane is needed, for example, for signal transduction or vesicle formation and fusion.^1−4^

A prominent example for protein–membrane interaction is human α-synuclein (αS), a protein that is highly abundant in neuronal cells, but its whole physiological function is still not completely unequivocally elucidated today.^5,6^*In vitro*, upon binding to lipid membranes, the N-terminus of αS undergoes a significant conformational transition with respect to its monomeric intrinsically disordered form, with some regions adopting a high level of α-helical structure.^7−13^

As the function of αS is not known, and membrane binding was confirmed only *in vitro* or indirectly in cells *via* colocalization^14^ to date, it is essential to get more insights into the native behavior of αS inside living cells. Recent in-cell EPR and NMR studies found αS being disordered in the cell.^15,16^ However, because of limited sensitivity, both studies could not exclude that approximately 20% of αS might be in a different conformational state in the cell. At the same time, αS membrane affinity and the extended membrane surface area inside cells suggest that the remaining undetected fraction might be in a membrane-bound state.

There exist various methods to investigate protein–membrane interaction *in vitro*,^17^ but studies in cells remain challenging because of the high background of the cellular components. Different microscopy techniques as well as magnetic resonance spectroscopy can circumvent this problem by using specific labels. While microscopy is exceedingly sensitive even at very low concentrations, membrane interaction is not measured directly but determined on the basis of colocalization in the cell.^17^ Nuclear magnetic resonance (NMR) and electron paramagnetic resonance (EPR) spectroscopy directly sense the effect of the membrane on the labeled protein but rely on higher concentrations.^15,16^ In NMR only the unbound fraction is visible and membrane-binding is followed by the disappearance of signals. In contrast, EPR directly measures the influence of the membrane on the attached label and thus enables detection of the bound and unbound state at the same time.^16,18^ Many reports show that EPR spectroscopy in combination with site-directed spin labeling (SDSL) is a suitable method to investigate protein–membrane interactions.^19−29^ Nitroxide spin labels are sensitive to local dynamics and chemical environment of the labeling site,^24,30−37^ and by analyzing the spectral shape, membrane binding can be followed (Figure 1).

**Figure 1:**
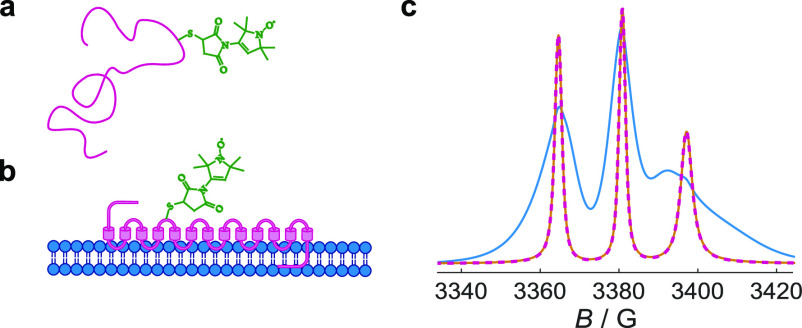
Schematic drawing of αS (pink) (a) in solution and (b) bound to a lipid membrane (blue). M-proxyl label for RS EPR experiment is represented by its structural formula (green; not to scale). (c) Simulated^4^ absorption EPR spectra of M-proxyl labeled αS A27C based on spectral fitting of continuous wave (CW) EPR measurements^27^ for αS in the absence of lipids (dashed line, pink) and in the presence of negatively charged lipid vesicles (blue) and uncharged lipid vesicles (yellow, overlaying with the pink spectrum).

Because sensitivity is a crucial point when investigating αS–lipid interactions in the cell,^15,16^ we set out to apply rapid-scan (RS) EPR spectroscopy. Comparison of continuous wave (CW) and RS EPR spectroscopy on an aqueous nitroxide sample revealed an improved signal-to-noise ratio (SNR) for RS EPR by a factor of 2.7 under our experimental conditions (Figure S1). The observed SNR gain afforded by RS EPR detection is likely from applying a higher microwave B1 on the sample without noticeable saturation effects. We note that the choice of optimal incident power for both RS and CW EPR is determined by spin relaxation time and sample heating, and therefore, the gains in SNR are expected to differ from sample to sample.^38^

Exploiting conventional CW EPR, it was shown previously *in vitro* that for spin-labeled αS, protein–membrane interaction,^39^*e.g.*, in the presence of negatively charged large unilamellar vesicles (LUVs), results in reduction of the rotational mobility of the spin label.^20,27^ This is reflected by spectral broadening.^40^ In the presence of uncharged vesicles, where no interaction between αS and the membrane is expected, the spectrum remains unchanged with respect to the spectrum in the absence of vesicles.

Prior to experimental investigations, RS EPR spectra were simulated based on the results of CW EPR studies^27^ (Figure 1c). For the experiments of the current study, we used 3-maleimido-PROXYL (M-proxyl) labeled αS A27C and the negatively charged 1-palmitoyl-2-oleoyl-*sn*-glycero-3-(phospho-rac-(1-glycerol)) (POPG) for preparation of LUVs (100 nm, Figure S2) as described in Robotta *et al.*(27) The uncharged lipid 1-palmitoyl-2-oleoyl-*sn*-glycero-3-phosphocholine (POPC) was used as a negative control.

As recently described, deletion of amino acids 2–11 in the αS primary sequence (αS Δ2–11) leads to reduced lipid binding.^27^ We used this αS variant without changing the position for SDSL as another negative control *in vitro* and in cells. We applied RS EPR spectroscopy to observe spectral changes upon protein–vesicle binding *in vitro*. Then, we tested the use of RS EPR for in-cell spectroscopy by microinjection of the αS variants either in the absence of vesicles or bound to POPG LUVs into oocytes of *Xenopus laevis* as a well-established model system for in-cell NMR and EPR^41−44^ and for in-cell characterization of αS.^15^ Next, we microinjected vesicles and protein successively to study the process of αS–lipid interaction inside the cell.

*In vitro*, we recorded the RS EPR spectra of the two M-proxyl labeled αS variants αS A27C and αS A27C Δ2–11 in the absence or presence of LUVs composed of different lipids (Figure 2). The spectra of αS showed a distinct broadening in the presence of POPG LUVs but not in the presence of POPC LUVs or absence of vesicles (Figure 2a), which is in agreement with simulated data^26^ (Figure 1c) and CW EPR spectroscopy (Figure S3a). Because membrane binding of αS goes along with an α-helical change of the secondary protein structure, these results were confirmed by CD spectroscopy (Figure S3b). The M-proxyl labeled control variant αS A27C Δ2–11 with reduced binding ability did not show significant spectral changes (Figures 2b and S2c,d).

**Figure 2:**
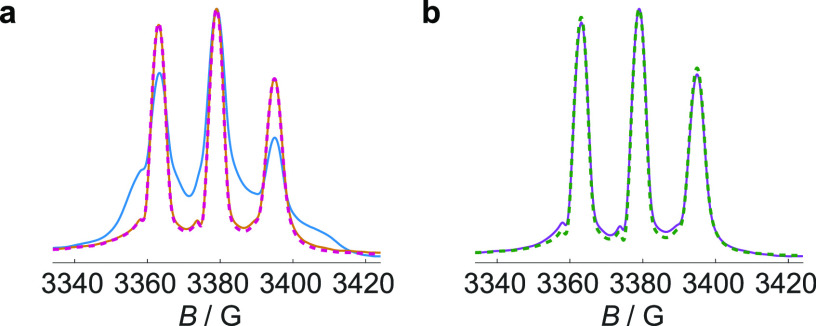
Experimental RS EPR spectra *in vitro*. (a) RS EPR of M-proxyl labeled αS A27C in the presence of negatively charged POPG LUVs (blue) compared to the presence of POPC LUVs (yellow), or in absence of vesicles (dashed line, pink). (b) Measurement of M-proxyl labeled αS A27C Δ2–11 in the presence of POPG LUVs (violet) compared to the absence of vesicles (dashed line, green). All spectra were normalized to the spectral maximum.

While αS–vesicle interaction has been demonstrated *in vitro* before,^26,27,45^ observation of the binding process in cells was limited by poor SNR.^15^ In order to prove the applicability of RS EPR to in-cell measurements, we microinjected M-proxyl labeled αS A27C either in the absence of vesicles or together with POPG or POPC LUVs into oocytes. Similar to the *in vitro* measurements, significant spectral broadening was found only in the presence of negatively charged lipids (Figure 3a). However, the effect appears to be slightly stronger *in vitro* than in cells (Figure S4). The spectral shape of vesicle-bound labeled protein does not change over time, indicating that the binding equilibrium seems to be stable in the context of the cell (Figure S5). After the measurement, preservation of oocyte integrity was checked visually, in order to exclude that the measurement was acquired in lysate instead of whole cells (Figure S6).

**Figure 3:**
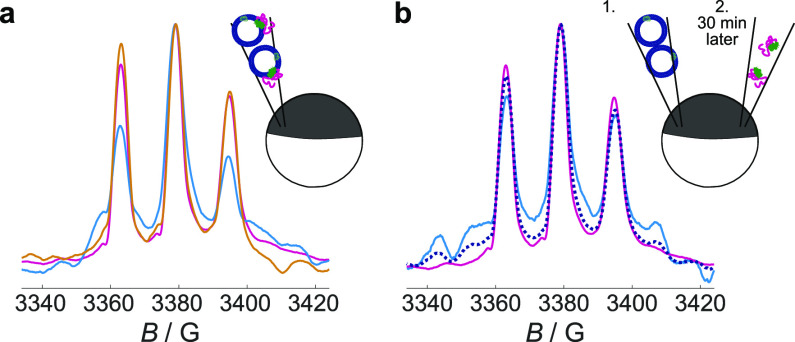
RS EPR spectra of M-proxyl labeled αS A27C. (a) Vesicle binding was achieved by *in vitro* incubation of αS with LUVs for 30 min prior to microinjection into oocytes. In-cell spectra of αS in the presence of POPG LUVs (blue) or POPC LUVs (yellow) compared to the in-cell spectrum upon microinjection of protein without lipids (pink). (b) Lipid vesicles were microinjected into oocytes. Thirty min later, αS was injected into the same cells. Spectrum in the presence of POPG LUVs of the first 10 min interval of the measurement (dashed line, dark blue, 7 min after injection) compared to the third 10 min interval (bright blue) and the in-cell spectrum of protein alone (pink).

To monitor the process of membrane binding in cells, we microinjected POPG LUVs into oocytes, incubated them for 30 min to ensure diffusive distribution within the cell,^15^ and performed a second microinjection with M-proxyl labeled αS A27C on the opposite side in the black hemisphere of the oocyte. The comparison of the spectrum obtained in the first 10 min interval of the measurement and the third 10 min interval (Figure 3b, dark and light blue) shows spectral broadening of the latter. This observation suggests that we were able to detect protein–lipid interaction in cells. However, the shape obtained from RS EPR in the first 10 min of acquisition was already broadened compared to the RS spectrum obtained from injection of only αS (Figure 3b, dark blue and pink) because the first scan was acquired 7 min after protein injection because of experimental procedures such as sample mounting and tuning.

Fast acquisition of RS EPR combined with spectral accumulation in data post processing allows varying the SNR and time resolution. We reduced the sampling time to 4.2 min (Figure S7) and quantified spectral changes in a time-resolved manner.

In order to describe the spectral changes, we introduced a semiempirical parameter, the protein–lipid interaction factor ξ (Figure 4; for details and error bars, see the Supporting Information). For the simulated spectra, protein–membrane interaction factors of ξ = 0.17 (fraction of membrane bound αS *b* = 0^27^) and ξ = 0.87 (*b* = 1^27^) were found. Significant changes of ξ in the presence of lipid vesicles were found for POPG but not for POPC LUVs for *in vitro* and in-cell experiments (Figure 4a). Spectral simulations enable conversion of ξ into the fraction of bound protein *b* for αS A27C (Figure S8).

**Figure 4:**
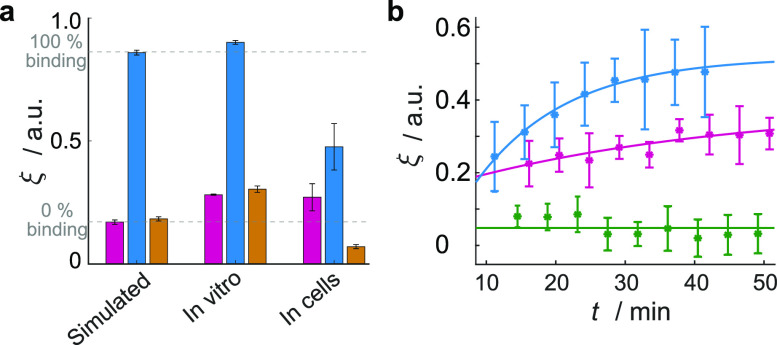
(a) Protein–lipid interaction factors (ξ) for αS in absence of LUVs (pink), or in the presence of POPG (blue) or POPC (yellow) LUVs corresponding to the spectra shown in Figures 1–3 A. (b) Quantification of vesicle binding in the cell by calculation of ξ in a time-resolved manner. Microinjection of αS A27C in oocytes with addition of POPG LUVs (blue) compared to the absence (pink) of artificial vesicles and microinjection of A27C Δ2–11 (green). Empirical fits (Table S3) were applied as a guide to the eye.

A clear signal broadening was also obtained for separate injection of αS and POPG LUVs (Figure 4b). Moreover, quantification of the intracellular binding process revealed saturation around 40 min after microinjection. The kinetics of spectral changes had to be ascribed to various effects such as protein diffusion within the cell and lipid binding and was approached with an asymptotic function (Table S3). Saturation was found for ξ = 0.52, which corresponds to *b* = 0.5. Interestingly, the spectrum obtained from αS A27C without vesicles exhibited also slight spectral changes and was also described with an asymptotic function. Nevertheless, no significant spectral change was found upon microinjection of αS A27C Δ2–11, and ξ(*t*) was described only by a constant value. This finding might be an indication for αS interaction with endogenous membranes or other macromolecules. Differences of ξ(0) between αS A27C with or without vesicles were within the error bars; the variation in ξ(0) of αS A27C Δ2–11 results from spectral differences within the protein mutants.

Former in-cell EPR and NMR studies on αS did not detect protein interaction with endogenous membranes.^15,16^ However, because they were limited by SNR and nitroxide signal reduction in the cell, they could not exclude a possible membrane binding of a small fraction of αS. Although nitroxide signal reduction to 1/2 *I*(*t*_0_) within 23 min was observed in our studies (Figure S9), RS EPR spectra indicate a small fraction of protein interacting with membranes in the context of a living cell. In order to exclude that this effect results from more ready reduction of solvent-exposed residues compared to membrane-bound ones, ascorbate reduction of POPG-bound labeled αS A27C was performed *in vitro*. All spectral components were reduced to a similar extent, thus confirming the intracellular protein–lipid interaction (Figure S10).

These results encourage the use of RS EPR for further investigations aiming to quantify the percentage of αS interacting with endogenous membranes upon delivery into a living cell. Furthermore, protein delivery into mammalian cells, such as non-neuronal A2780 and HeLa cells and neuronal B65, SK-N-SH, and RCSN-3 cells,^16^ might be another step on the way to characterize the physiological role of αS and other membrane-binding proteins.

In this work, we used RS EPR to study protein–membrane interactions of αS and negatively charged lipid membranes. We showed that RS EPR spectroscopy enables detection of protein–vesicle interaction *in vitro* and in oocytes of *Xenopus laevis*. Moreover, we were able to monitor the process of vesicle binding to artificial and endogenous lipid membranes in the cell. The absence of spectral broadening in an αS variant with limited binding ability supports the hypothesis that RS EPR spectroscopy possesses the potential to observe small fractions of protein being bound to endogenous membranes.

## Experimental Methods

Detailed information on sample preparation, spectroscopy experiments, and data analysis is given in the Supporting Information. Briefly, αS variants were expressed in *E. coli* and purified *via* anion exchange. Spin-labeling was performed with M-proxyl, and the remaining label was removed *via* buffer exchange to 10 mM Tris-HCl pH 7.4 containing 150 mM NaCl.

*X. laevis* oocytes on stage V/VI were obtained from EcoCyte Bioscience. Either 50 nL of protein (1.2 mM) or protein (1.2 mM) preincubated for 30 min with 70 mM LUVs was microinjected into the black hemisphere of ocytes using a Nanoject III (Drummond; Broomall, PA). For investigation of intracellular LUV binding kinetics, the LUV solution (100 mM) was injected into each oocyte. After an incubation time of 30 min, the αS solution was microinjected into the opposite side within the black hemisphere.

Seven microinjected oocytes were collected in a Q-band tube and mounted into the resonator of the spectrometer. Rapid-scan spectra were recorded using an Elexsys 500 X-band spectrometer equipped with the Rapid-Scan Accessory (Bruker). Sinusoidal rapid magnetic-field scans at a frequency of 20 kHz with a scan width of 200 G were applied using a 2D field versus delay experiment with 270 slices, each lasting 10 s. For quantitative analysis 24 scans were added resulting in a time resolution of 4.18 min, otherwise 56 scans were added for a time resolution of 10 min. Spectra were smoothed and background corrected as described in the Supporting Information.

The protein–membrane interaction factor (ξ) was introduced in order to quantify membrane binding as follows:
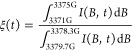
Error bars were determined as follows:
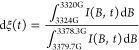


## Supporting Information Available

Experimental details, sample preparation, supplementary figures, and list of abbreviations (PDF)
