# Supplementary material for: Intracellular Protein–Lipid Interactions Studied by Rapid-Scan Electron Paramagnetic Resonance Spectroscopy
Source: J Phys Chem Lett. 2021 Mar 5;12(9):2471–5. doi: 10.1021/acs.jpclett.0c03583 (PMC7957861; doi:10.1021/acs.jpclett.0c03583)
Supplement: Supplementary file 1 — jz0c03583_si_001.pdf [file jz0c03583_si_001.pdf]

Supporting information for:

Intracellular Protein−Lipid Interactions Studied by Rapid-Scan Electron Paramagnetic Resonance Spectroscopy

Theresa S. Braun,[†, ‡] *Juliane Stehle,*[†, ‡] *Sylwia Kacprzak,*[#] *Patrick Carl,*[#] *Peter Höfer,*[#] *Vinod Subramaniam,*[§] *and Malte Drescher*[*,†]

[†] Department of Chemistry and Konstanz Research School Chemical Biology, University of Konstanz, 78457 Konstanz, Germany

[#] Bruker BioSpin GmbH, Silberstreifen 4, 76287 Rheinstetten, Germany

[§] Vrije Universiteit Amsterdam, De Boelelaan 1105, 1081 HV Amsterdam, The Netherlands

[‡]These authors contributed equally.

E-mail: malte.drescher@uni-konstanz.de

Table of Contents

A. Sample preparation S3

Expression, purification and labeling of αS S3

Preparation and characterization of large unilamellar vesicles (LUVs) S4

Microinjection S5

B. Spectroscopy S6

Determination of the signal-to-noise ratio (SNR) S6

Spectral simulations S6

Rapid-scan EPR S7

Continuous wave EPR S7

Circular dichroism S7

C. Data analysis S8

Continuous wave EPR S8

Rapid-scan EPR S8

Processing of raw spectra S8

Quantification of the binding process S9

D. Supplementary figures S10

E. List of Abbreviations S16

F. References S17

A. Sample preparation

Expression, purification and labeling of αS

α-synuclein (αS) was prepared recombinantly as recently described.[1] Briefly, plasmid pT7-7_SNCA_A27C was generated *via* site-directed mutagenesis based on pT7-7 asyn WT (addgene). pET11C_ASYN_Δ2–11 was kindly provided by the Leist Lab[2-3] and a cysteine was introduced in full analogy to pT7-7_ASYN_A27C with just 10 amino acids lacking in the N-terminus.

Plasmids were transformed into *E. coli* cells as follows. Chemically competent BL21-Gold (DE3) *E. coli* cells were thawed on ice, mixed with the respective plasmid, and incubated on ice for 30 min, before being heat-shocked at 42 °C for 1 min with subsequent incubation on ice (2 min) and being rescued in pre-warmed (37 °C) Super Optimal Broth with carbolite repression (S.O.C.) medium for 1 h at 37 °C and 140 rpm. Transformed cells were grown on LB-agar plates (Lennox) containing 50 µg/mL carbenicillin at 37 °C and 180 rpm overnight. For glycerol stock creation, a single colony was picked for inoculation of 10 mL LB-medium containing 50 µg/mL carbenicillin, grown overnight at 37 °C and 180 rpm, mixed with 50 % (v/v) glycerol, shock-frozen in liquid nitrogen, and stored at -80 °C.

1 L LB medium containing carbenicillin (50 µg/mL) was inoculated with 1 % overnight culture of BL21-Gold(DE3) *E. coli* (Agilent) containing plasmid pET11C_ASYN_Δ2–11. At OD_{600} = 0.8, protein expression was induced in the presence of 1 mM IPTG. After 5 h of incubation at 37 °C and 180 rpm shaking, expression was stopped *via* centrifugation. Lysis was performed in 25 mL Tris-HCl (10 mM, pH 7.4) in the presence of 1 mM EDTA, 1 mM phenylmethylsulfonylfluoride (PMSF, protease inhibitor) and 1 mM dithiothreitol (DTT) with a Q700 SONICATOR (QSONICA, equipped with a 1.6 mm tip probe, 2 min cycling with 1 s pulse-on and 1 s pulse-off, amplitude 10). Nucleic acids were removed *via* streptomycin sulfate precipitation (1 % w/v), and proteins were isolated *via* ammonium sulfate precipitation. Purification of αS was performed on an ÄKTAprime plus chromatography system equipped with an anion exchange column (RESOURCE Q) (GE Healthcare Life Sciences). Fractions containing αS were pooled and dialyzed against 10 mM Tris, pH 7.4 containing 1 mM DTT to prevent cysteine dimerization. Protein concentrations were determined at a BioPhotometer® D30 (Eppendorf) at 280 nm.

Prior to labeling, DTT was removed using PD-10 desalting columns (GE Healthcare). A 6x molar excess of 3-maleimido-2,2,5,5-tetramethyl-1-pyrrolidinyloxy (M-proxyl, Sigma-Aldrich Chemie GmbH) was added and the protein solution was incubated overnight at 4° C. Subsequently, free label was removed with Microsep™ Advance 3K MWCO filters at the same time as the buffer was exchanged to 10 mM Tris-HCl pH 7.4 containing 150 mM NaCl. The protein solution was concentrated in Amicon Ultra-0.5 centrifugal filter units (3K) to a concentration of about 3.5 mM (Table S1). Spin concentration was determined at an EMXnano benchtop X-band spectrometer (Bruker Biospin), and the labeling efficiency was calculated as the quotient of spin and protein concentration. The obtained labeling efficiency was in the range of 90-100 %. Samples were kept on ice during the washing process to minimize aggregation and stored at -80 °C afterwards.

Table S1. Protein and spin concentrations of expressed αS variants.

αS variant	Protein concentration / mM	Spin concentration / mM	Labeling efficiency
**WT**	2.9	0	0 %
**A27C**	3.8	3.4	90 %
**Δ2–11 A27C**	3.5	3.5	100 %

Preparation and characterization of large unilamellar vesicles (LUVs)

1-palmitoyl-2-oleoyl-sn-glycero-3-phospho-(1'-rac-glycerol) (POPG) and 1-palmitoyl-2-oleoyl-sn-glycero-3-phosphocholine (POPC) were purchased from Sigma-Aldrich, solved in chloroform, dried to thin films, resuspended in Tris-HCl (10 mM, pH 7.4) containing 150 mM NaCl, followed by five cycles of freezing (liquid nitrogen) and thawing (37 °C; water bath). Extrusion was performed using 100 nm polycarbonate membranes (GE Healthcare). The final lipid concentration was 100 mM.

Vesicle size was checked by dynamic light scattering (DLS, Figure S2). 1 µL of the LUV solution was added to 1 mL Tris-HCl (10 mM, pH 7.4) containing 150 mM NaCl in a 1 cm polycarbonate cuvette. The sample was measured at 20 °C using a Zetasizer Nano-ZS spectrometer equipped with a 4 mW He-Ne laser (vertically polarized incident radiation of 633 nm wavelength, Malvern Panalytical).

Microinjection

Microinjection was performed as previously described.[4-5] Briefly, *Xenopus laevis* oocytes on stage V/VI (EcoCyte Bioscience, Caustrop-Rauxel) were kept in modified Barth's saline (MBS, 88 mM NaCl, 1 mM KCl, 0.4 mM $CaCl_2$, 0.33 mM $Ca(NO_3)_2$, 0.8 mM $MgSO_4$, 5 mM Tris-HCl, 2.4 mM $NaHCO_3$) at 18 °C. Either 50 nL of protein (1.2 mM) in Tris-HCl buffer (10 mM, pH7.4, 150 mM NaCl) or protein (1.2 mM) preincubated for 30 min in Tris-HCl buffer (10 mM, pH7.4, 150 mM NaCl) containing 70 mM LUVs was microinjected into the black hemisphere of oocytes using a Nanoject III automatic nanoliter injector with fitting micromanipulator MM33 (Drummond; Broomall, PA). For investigation of LUV binding kinetics, co-injection was performed as follows: First, the LUV solution (100 mM) was injected into each oocyte. After an incubation time of 30 min at 18 °C, the αS solution was microinjected into the opposite side within the black hemisphere. Detailed concentrations and volumes are given in Table S2. Subsequently, seven microinjected oocytes were collected in a Q-band tube (quartz glass, 1 mm inner diameter, Bruker), and mounted into the resonator of the spectrometer.

Table S2. Volumes and concentrations used for co-injections into oocytes.

αS variant	Protein concentration / mM	Protein injection volume / nL	Lipid concentration LUVs / mM	Lipid injection volume / nL	Lipid / protein ratio
**A27C**	3.8	15	100	35	60
**Δ2–11 A27C**	3.5	16.2	100	33.9	60

B. Spectroscopy

Determination of the signal-to-noise ratio (SNR)

Quantitative SNR comparison between RS and CW EPR was performed using an EMXplus (Bruker) equipped with a Super High QE (SHQE) resonator (Bruker) and Elexsys 500 X-band spectrometer (Bruker) equipped with the Rapid-scan (RS) accessory (Bruker). 4.04 µM TEMPOL was dissolved in water, and the solution was degassed and loaded into glass capillaries with 1 mm inner diameter (HIRSCHMANN® ringcaps®). A power sweep was performed for both techniques. (Figure S1a) Nitroxide saturation was found at 1.26 mW or 100 mW for CW or RS. Accordingly, for CW experiments, a power of 1.26mW was chosen. For RS experiments, the power was limited to 20mW to avoid heating effects (see section rapid-scan EPR) respectively.

For CW spectroscopy, the experimental parameters were optimized to a modulation amplitude of 1 G at a modulation frequency of 100 kHz. One scan of 200 G was acquired in 10 s with 1200 points and a time constant of 0.01 ms. Quality factors were 10000 for the empty resonator and 1800 for the aqueous nitroxide sample. A SNR ratio of 8.9 was determined. (Figure S1b)

RS spectroscopy was performed with sinusoidal rapid magnetic-field scans at a frequency of 20 kHz with a scan width of 200 G at a scan rate of 12.6 MG/s at 1133 points. One scan was acquired in 10 s and consisted of 200400 averages. Quality factors were 5000 for the empty resonator and 1300 for the aqueous nitroxide sample. A pseudo-modulation of 1 G was applied to reveal the first-derivative spectrum and calculate the SNR. A SNR of 24 was determined. (Figure S1c) SNR comparison was performed on the first-derivative spectra, only, since integration changes the frequency distribution of the noise.[6]

Spectral simulations

Absorption spectra were simulated with the use of Matlab R2019b and the toolbox EasySpin 5.2.25.[7] Simulation parameters were obtained from Robotta et al..[8] For M-proxyl labeled αS A27C in the absence of lipid vesicles we used A = [13 13 110.16] MHz, g = [2.00906 2.00687 2.003], lw = 0.11 mT, and τ_r = 0.43 ns. In the presence of vesicles, an additional slower component with A = [13 13 107.34] MHz and τ_r = 3.06 ns was applied for simulation of two-component spectra. For POPG LUVs, the amount of the slow component was 98 %, for POPC LUV 2 %.

Rapid-scan EPR

Either 50 µL aqueous solution or 7 oocytes were loaded into Q-band tubes (quartz glass, 1 mm inner diameter, Bruker) and rapid-scan spectra were recorded using an Elexsys 500 X-band spectrometer (Bruker) equipped with the rapid-scan (RS) Accessory (Bruker). The RS Accessory comprises: (i) water-cooled RS coils mounted on a 10'' magnet for modulating the magnetic field, (ii) RS coil driver to generate the waveform for the scan and control the current of the RS coils, (iii) a capacitor circuit for resonant mode operation, (iv) the microwave front end for detection and amplification of the EPR signal, (v) the RS acquisition unit for signal digitalization, and (vi) dedicated RS resonator that is transparent to the rapidly changing magnetic fields thus avoiding generation of eddy currents.

Rapid-scan spectra were acquired applying sinusoidal rapid magnetic-field scans at a frequency of 20 kHz with a scan width of 200 G (scan rate of 12.6 MG/s). The scan was centered at a static magnetic field of 3280 G with a microwave power of 20 mW. Temperature stability was achieved with a nitrogen flow and temperature stability was ensured by monitoring the stability of the AFC during tuning. It was stable at experimental attenuation which indicates that the resonator and sample were not heated. Data was acquired using a 2D field vs. delay experiment with 270 slices and a minimum delay between slices. Each field slice was acquired in 10 s and consisted of 200400 averages.

Continuous wave EPR

In vitro samples were loaded into glass capillaries with 1 mm inner diameter (HIRSCHMANN® ringcaps®) and measured on an EMXnano benchtop X-band spectrometer (Bruker Biospin) (9.637 GHz) with optimized parameters. (Figure S11) using a microwave power of 3.162 mW, a modulation amplitude of 0.8 G at a modulation frequency of 100 kHz at room temperature.

Circular dichroism

The Circular dichroism (CD) spectra were measured with a JASCO J-715 spectropolarimeter at 20 °C. Protein samples (20 µM) were mounted in 0.5 mm demountable cuvettes. Baseline correction and subtraction of the background spectrum (buffer without protein) were applied to the raw data. CD data measured at a HT value above 550 V were removed, as the signal got noisy and unreliable.

C. Data analysis

Continuous wave EPR

Spectra were corrected according to the microwave frequency, baseline-corrected, and normalized to the double integral with the help of Matlab 2019b. The measured spectra were analyzed using Matlab R2019b (The MatWorks, Inc. Natrick,MA). Field-modulated EPR spectra were computed with the toolbox EasySpin 5.2.25.[7] All spectra were baseline-corrected with a second order polynomial function, smoothed using a Savitzky-Golay filter with a second order polynomial and a frame length of 51. Depending on the spin concentration, 10 to 30 scans were accumulated and normalized to the double integral.

Rapid-scan (RS) EPR

Processing of raw spectra

The detected time domain EPR signal was processed on board into the field domain with a baseline correction to account for the driving waveform. No deconvolution of the signal was necessary as the signals were not in the RS regime. The time domain axis was directly transformed into the field domain.

Addition of individual time slices was performed in order to optimize the signal-to-noise ratio (SNR). For quantitative analysis 24 scans were added resulting in a time resolution of 4.18 min, otherwise 56 scans were added for a time resolution of 10 min. Spectra were smoothed using a Savitzky-Golay filter with a second polynomial order and a frame length of 53. For the in-vitro experiments, the spectrum of unlabeled αS was used for background correction, for the oocyte experiments, spectra acquired after nitroxide reduction were used. Due to changes in microwave-frequency, Q-factor and measurement artifacts affecting the baseline, the reference backgrounds (bg(B)) were corrected ($\mathrm{bg_{corr}}(B)$) with three parameters in an iterative way to minimize the baseline difference in the field ranges of 3283 G to 3355 G and 3398 G to 3475 G for each sampling frame individually:

$$\mathrm{bg_{corr}}(B) = a \cdot [\mathrm{bg}(B) - (b + c \cdot B)]$$

After background-subtraction (I(field) - $\mathrm{bg_{corr}}$(field)), the magnetic field-axis was recalculated to the microwave-frequency of 9.493204 GHz, and the spectra were normalized to the maximum amplitude of the center field peak.

Quantification of the binding process

As revealed from Figure 1b, 2a, and 3, spectral broadening can most prominent be seen in the low-field peak. Therefore, the integral of the inter-peak space between lowfield and centerfield peak (3371-3375 G) was chosen to calculate a protein–lipid interaction factor ξ, the area below a baseline interval (3320-3324 G) represents effects caused by noise and was used to calculate error bars. To compensate for nitroxide reduction effects, normalization to the area below the centerfield peak (3378.3-3379.7 G) was performed (Figure S5).

$$\xi(t) = \frac{\int_{3371\,\mathrm{G}}^{3375\,\mathrm{G}} I(B,t)\, dB}{\int_{3379.7\,\mathrm{G}}^{3378.3\,\mathrm{G}} I(B,t)\, dB}$$

In order to reveal intracellular lipid binding with small impact on the spectral shape, time-resolved measurements (Figure 4b) were fitted with the following function:

$$\xi(t) = a - (a - b) \cdot \exp(-c \cdot t)$$

Resulting parameters are given in table S3.

Table S3. Parameters used for fitting of the time-resolved protein–lipid interaction factors in the cell. (Figure 4b)

Injected sample	a	b	c
**αS A27C**	0.385	0.143	0.0252
**POPG & αS A27C**	0.519	-0.138	0.0761
**αS Δ2–11 A27C**	0.0492	0	∞

D. Supplementary figures

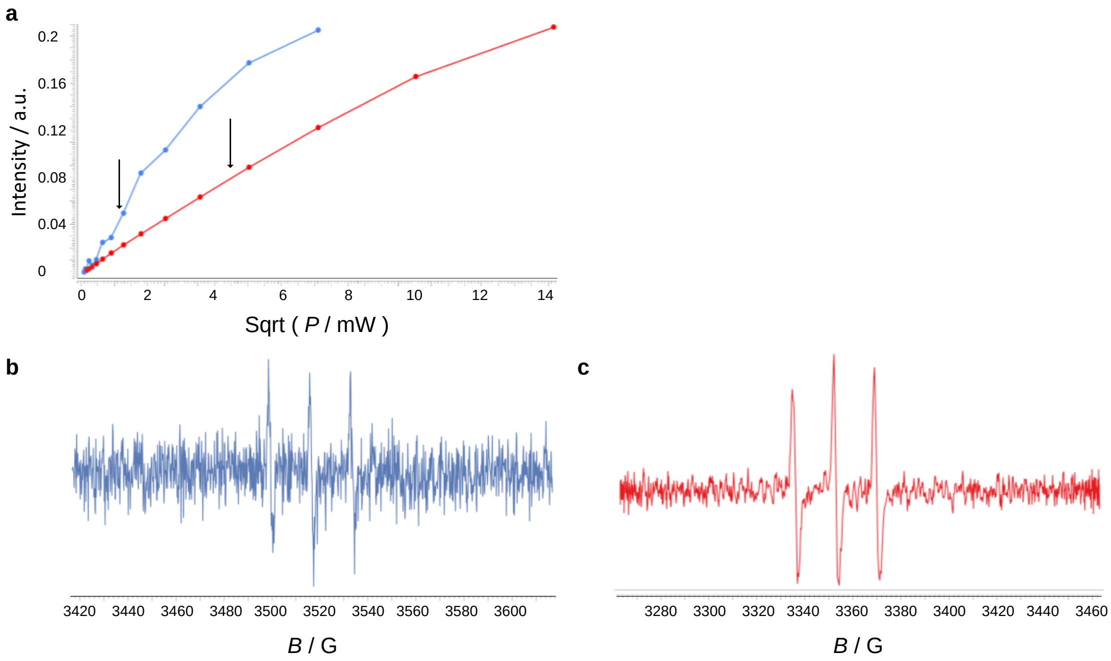

Figure S1. SNR comparison between RS and CW EPR on degassed TEMPOL in water (4.04 µM). (a) Saturation curve for RS (red) and CW (blue). Arrows indicate the microwave powers that were chosen for the SNR comparison. (b) Optimized CW EPR experiment at 1.26 mW microwave power, SNR=8.9. (c) RS EPR experiment at 20 mW microwave power upon pseudo-modulation to obtain the first-derivative spectrum. A SNR of 24 was determined.

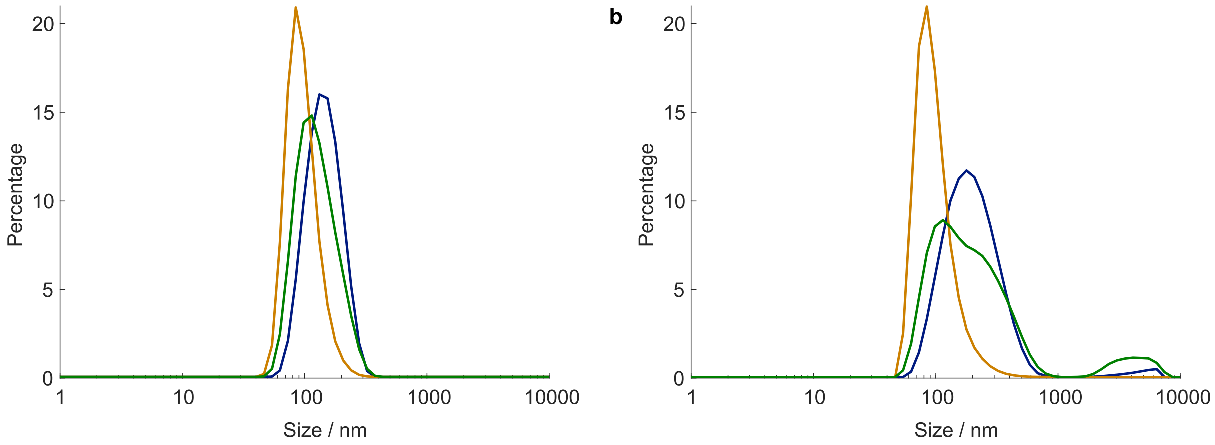

Figure S2. DLS analysis of the POPG (a) and POPC (b) LUVs used in this study. The vesicle size of 100 nm was confirmed by the three parameters intensity (blue), volume (green) and number (yellow).

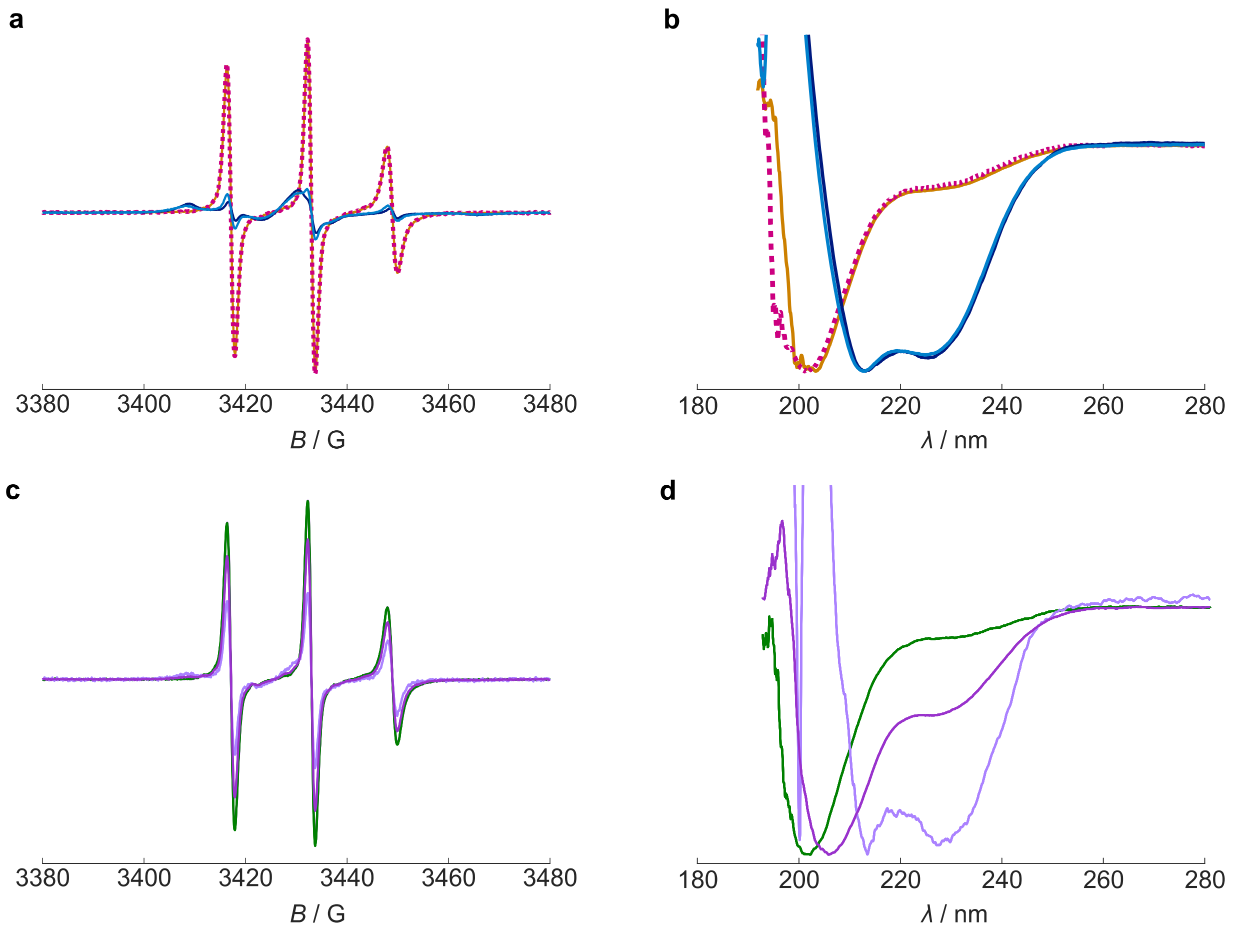

Figure S3. CW EPR (a&c) and CD spectra (b&d) of M-proxyl labeled αS A27C (a&b), and αS A27C Δ2–11 (c&d) in buffer (pink dashed line or green), in POPC solution (protein:lipid = 1:60, yellow, overlaying with the pink dashed spectrum in a), or in POPG solution (protein:lipid = 1:60, light blue or light violet; protein:lipid = 1:600, dark blue or dark violet). Vesicle binding and its conformational switch to α-helical structures is indicated by spectral broadening of the EPR spectra and the shift of the dip in the CD spectrum from 199 nm to a double dip at 209 and 222 nm.

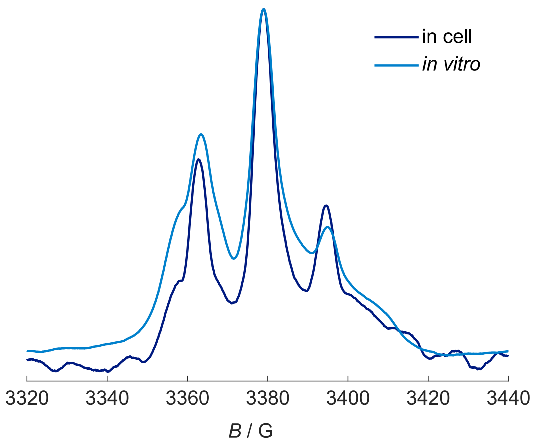

Figure S4. Comparison of M-proxyl labeled αS A27C spectra that were incubated with POPG LUVs and either measured in buffer (bright blue) or injected into oocytes (dark blue). Repeatable smaller peaks outside the three line nitroxide in-cell spectrum are artifacts that result from resonator instabilities.

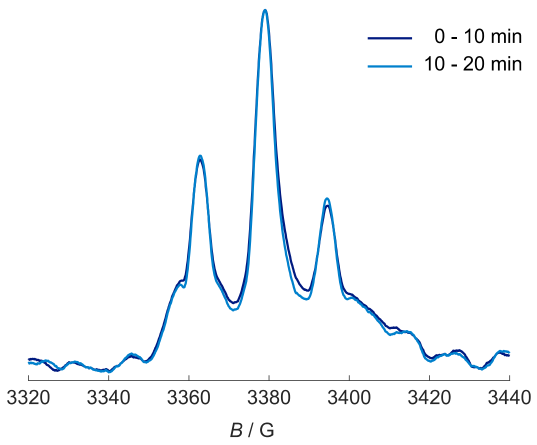

Figure S5. Time-resolved spectra of M-proxyl labeled αS A27C that was incubated with POPG LUVs prior to injection into oocytes. As indicated by the spectra that do not change in time, the binding equilibrium obtained *in vitro* does not change in the cell. Repeatable smaller peaks outside the three line nitroxide spectra are artifacts that result from resonator instabilities.

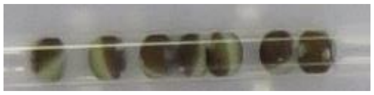

Figure S6. After the measurement, preservation of oocyte integrity was checked visually, in order to exclude that the measurement was acquired in lysate instead of whole cells.

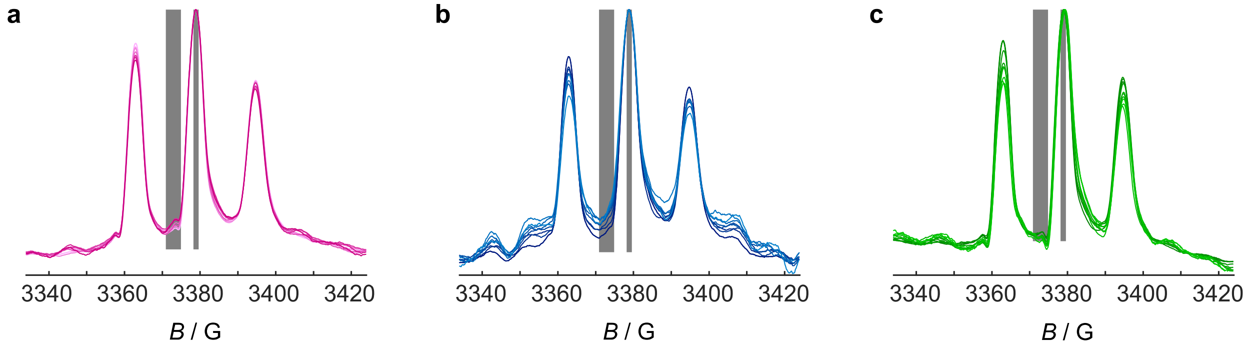

Figure S7. In-cell EPR spectra of αS A27C M-proxyl alone (a, light pink ─time→ dark pink), with POPG (b, dark blue ─ time → light blue), and αS Δ2–11 A27C M-proxyl (c, dark green ─ time → light green) that were used for calculation of ξ in figure 4b. Each spectrum corresponds to 4.2 min sampling time. Areas that were used for calculation of the protein−lipid interaction factor ξ were highlighted in grey. Repeatable smaller peaks outside the three line nitroxide spectra are artifacts that result from resonator instabilities.

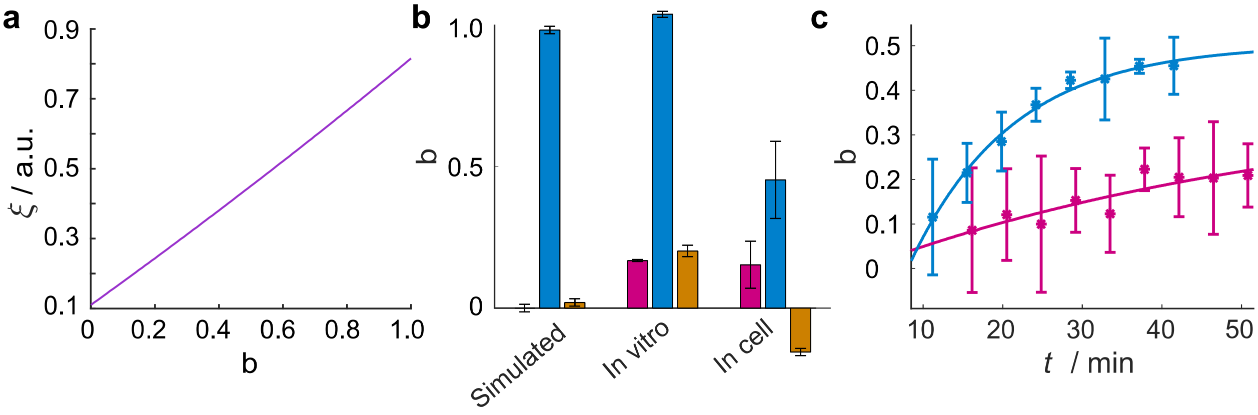

Figure S8. Conversion between the protein−lipid interaction factors ξ and the fraction of bound protein b. (a) Simulation of M-proxyl-labeled αS A27C spectra at different bound fractions b and subsequent calculation of ξ revealed the relation $\xi(\mathrm{b}) = 0.049\ \mathrm{b}^2 + 0.66\ \mathrm{b} + 0.17$. (b) Fraction of bound protein b for αS in the absence of LUVs (pink), or in the presence of POPG (blue) or POPC (yellow) LUVs. As indicated by the negative b value for POPC-bound M-proxyl-labeled αS A27C, absolute numbers have to be treated with caution since conversion was performed based on spectra simulated in situ. (c) Quantification of lipid binding in the cell by conversion of ξ. Microinjection of αS A27C in oocytes with addition of POPG LUVs (blue) compared to the absence (pink) of artificial vesicles. No reference data is available for M-proxyl labeled αS A27C Δ2–11, because this delete variant binds only very weakly to membranes. Therefore, translation of ξ into b is not possible for this negative control. Empirical fits (Table S4) were applied as guide to the eye.

Table S4. Parameters used for fitting of the lipid-bound fraction b of M-proxyl-labeled αS A27C in the cell over time (Figure S8c) with an exponential function: $\xi(t) = a - (a - b) \cdot \exp(-c \cdot t)$

Injected sample	A	b	c
**αS A27C**	0.384	-0.0147	0.0175
**POPG & αS A27C**	0.503	-0.439	0.0779

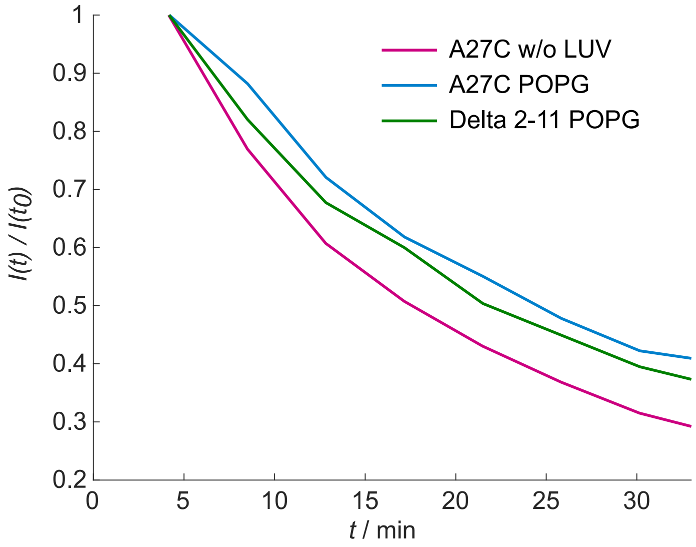

Figure S9. Nitroxide signal depletion as indicated by the intensity of the centerfield peak. Since the spectral shape did not change over time, not only the spectral integral but also the peak intensity was directly proportional to the number of spins. However, we decided for the intensity as a measure because it was less prone to errors caused by decreasing SNR.

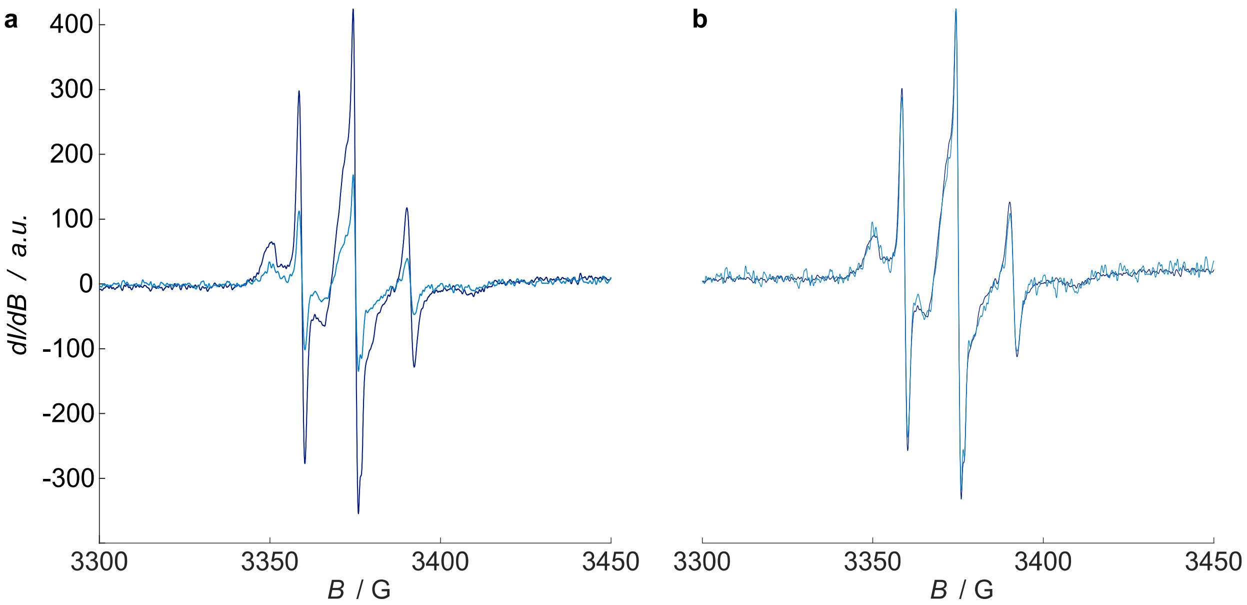

Figure S10. Ascorbate reduction assay. M-proxyl labeled αS A27C (222 µM) was incubated with POPG LUVs (13.3 mM) for 30 min. Then, ascorbate was added (6.6 mM in water, pH 7.4) and spectra were recorded subsequently after sample mounting (dark blue) and 30 min later (light blue). (a) The peak-to-peak amplitude was reduced from 779 a.u. to 303 a.u. within 30 min. (b) Normalization to the maximum amplitude of the spectra shows that the spectral shape did not change. This observation indicates that all spin labels are to the same extent prone to reduction.

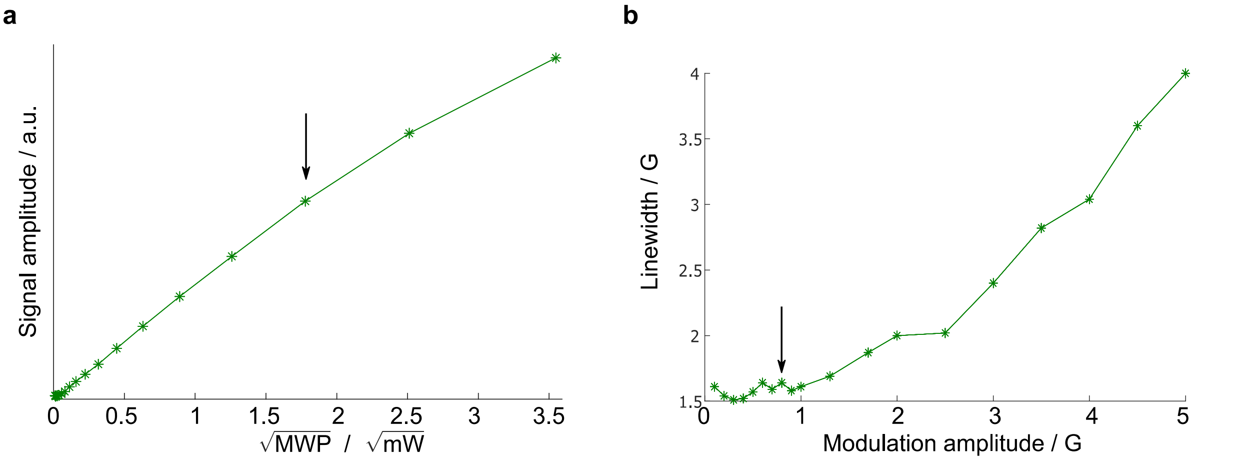

Figure S11. Optimization of the measurement parameters for nitroxides in aqueous solutions on the EMXnano. (a) A power sweep revealed saturation starting at around 6 mW microwave power (MWP). (b) A sweep of the modulation amplitude revealed linebroadening starting at 1 G. In order to exlude effect resulting from nitroxide saturation or line broadening, a MWP of 3.162 mW and a modulation amplitude of 0.8 G were chosen. (arrows)

E. List of Abbreviations

Abbreviation	Explanation
αS	α-synuclein
CD	Circular dichroism
CW	Continuous wave
DLS	Dynamic light scattering
DTT	Dithiothreitol
E. coli	*Escherichia coli*
EPR	Electron paramagnetic resonance
IPTG	Isopropyl β-D-1-thiogalactopyranoside
LUVs	Large unilamellar veislces
M-proxyl	3-Maleimido-2,2,5,5-tetramethyl-1-pyrrolidinyloxy
MWP	Microwave power
PMSF	Phenylmethylsulfonyl fluoride
POPC	1-palmitoyl-2-oleoyl-sn-glycero-3-phosphocholine
POPG	1-palmitoyl-2-oleoyl-sn-glycero-3-phospho-(1'-rac-glycerol)
RS	Rapid-scan
RT	Room temperature
SDSL	Site-directed spin labeling
SNR	Signal-to-noise ratio
S.O.C.	Super Optimal Broth with carbolite repression
WT	Wildtype
X. laevis	*Xenopus laevis*

F. References

1. Cattani, J.; Braun, T.; Drescher, M. Probing alpha-synuclein conformations by electron paramagnetic resonance (EPR) spectroscopy. In *Alpha-Synuclein: Methods and Protocols*; Bartels, T., Ed; Humana Press: New York, NY, 2019; pp 247–260.

2. Gerding, H. R.; Karreman, C.; Daiber, A.; Delp, J.; Hammler, D.; Mex, M.; Schildknecht, S.; Leist, M. Reductive modification of genetically encoded 3-nitrotyrosine sites in alpha-synuclein expressed in E.coli. *Redox. Biol.*. **2019,** *26*, 101251.

3. Robotta, M.; Hintze, C.; Schildknecht, S.; Zijlstra, N.; Jungst, C.; Karreman, C.; Huber, M.; Leist, M.; Subramaniam, V.; Drescher, M. Locally resolved membrane binding affinity of the N-terminus of alpha-synuclein. *Biochemistry*. **2012,** *51* (19), 3960–2.

4. Cattani, J.; Subramaniam, V.; Drescher, M. Room-temperature in-cell EPR spectroscopy: alpha-Synuclein disease variants remain intrinsically disordered in the cell. *Phys.. Chem.. Chem.. Phys.* **2017.** *19* (28), 18147-18151.

5. John, L.; Drescher, M. Xenopus laevis oocytes preparation for in-cell EPR spectroscopy. *Bio-protocol* **2018.** *8* (7), e2798.

6. Tseitlin, M.; Eaton, S. S.; Eaton, G. R. Uncertainty analysis for absorption and first-derivative EPR spectra. *Concepts Magn. Reson.* **2012.** *40A* (6), 295–305.

7. Stoll, S.; Schweiger, A. EasySpin, a comprehensive software package for spectral simulation and analysis in EPR. *J. Magn. Reson.* **2006.** *178* (1), 42–55.

8. Robotta, M.; Gerding, H. R.; Vogel, A.; Hauser, K.; Schildknecht, S.; Karreman, C.; Leist, M.; Subramaniam, V.; Drescher, M. Alpha-synuclein binds to the inner membrane of mitochondria in an alpha-helical conformation. *ChemBioChem* **2014.** *15* (17), 2499–502.
